# The Circadian *tau* Mutation in Casein Kinase 1 Is Part of a Larger Domain That Can Be Mutated to Shorten Circadian Period

**DOI:** 10.3390/ijms20040813

**Published:** 2019-02-14

**Authors:** Anandakrishnan Venkatesan, Jin-Yuan Fan, Samuel Bouyain, Jeffrey L. Price

**Affiliations:** Division of Molecular Biology and Biochemistry, School of Biological Sciences, University of Missouri—Kansas City, Kansas City, MO 64110, USA; avhd3@yahoo.com (A.V.); priceji@umkc.edu (J.-Y.F.); bouyains@umkc.edu (S.B.)

**Keywords:** circadian rhythms, PER protein, protein/protein interactions, protein phosphorylation, biological clocks, nuclear localization signal, phosphate recognition

## Abstract

Drosophila Double-time (DBT) phosphorylates the circadian protein Period (PER). The period-altering mutation *tau*, identified in hamster casein kinase I (CKIε) and created in Drosophila DBT, has been shown to shorten the circadian period in flies, as it does in hamsters. Since CKI often phosphorylates downstream of previously phosphorylated residues and the *tau* amino acid binds a negatively charged ion in X-ray crystal structures, this amino acid has been suggested to contribute to a phosphate recognition site for the substrate. Alternatively, the *tau* amino acid may affect a nuclear localization signal (NLS) with which it interacts. We mutated the residues that were close to or part of the phosphate recognition site or NLS. Flies expressing DBT with mutations of amino acids close to or part of either of these motifs produced a shortening of period, suggesting that a domain, including the phosphate recognition site or the NLS, can be mutated to produce the short period phenotype. Mutation of residues affecting internally placed residues produced a longer period, suggesting that a specific domain on the surface of the kinase might generate an interaction with a substrate or regulator, with short periods produced when the interaction is disrupted.

## 1. Introduction

Circadian rhythms are biological processes that are controlled by environmental cues and exhibit oscillations of 24 h during a typical diurnal cycle. Although these rhythms are controlled by the environment, in the absence of these cues, these oscillations persist with a period of approximately 24 h [1]. Analyses in both Drosophila and mammals have revealed that the clock mechanism is highly conserved between them. In both, the clock mechanism requires the oscillations of clock gene products and their control through both transcriptional and post-translational feedback loops. In Drosophila, the levels of Period (PER) and Timeless (TIM) proteins accumulate in the cytoplasm during the night, initially accumulating as a heterodimer and then translocating to the nucleus. Nuclear PER represses *per* and *tim* transcription by binding to a transcription factor complex of dCLOCK (dCLK) and dCYCLE (dCYC) proteins. The dCLK/dCYC complex activates transcription of *per*, *tim* and other genes that produce various outputs of the circadian clock. At the onset of day, the CRY photoreceptor protein responds to the light, and this targets TIM for degradation [2]. The Drosophila ortholog of casein kinase Iδ (CKIδ) and casein kinase Iε (CKIε), Doubletime (DBT), phosphorylates PER and targets it for degradation [3,4,5,6,7,8,9,10]. DBT plays a role in preventing the earlier accumulation of PER in the nucleus by targeting its degradation in the cytoplasm and also in relieving the repression of PER on its own transcription by targeting PER for degradation in the nucleus. Other kinases and phosphatases, including SHAGGY, casein kinase II, protein phosphatase 2A and protein phosphatase 1, also function together with DBT to contribute to the circadian mechanism [11,12,13,14,15,16,17,18].

Casein kinase 1 ε/δ is also a key regulator of PER phosphorylation and degradation in the mammalian circadian mechanism [19]. The period-altering mutations identified in mammalian CKI have been shown to shorten period [19,20], while the period-altering mutations identified in Drosophila DBT mostly lengthen period [5,6,7], suggesting initially that there was an evolutionary difference in the vertebrate and Drosophila CKI mechanism. However, the shorter periods of the *tau* and *dbt^S^* mutations have been shown to be evolutionarily conserved in transgenic flies expressing either Drosophila DBT or vertebrate CKIδ [21]. It has also been shown in both mammals and flies that complete loss of kinase activity of CKI through mutations of DBT or CKI (e.g., DBT^K/R^) [10,22] and tissue specific knockout or pharmacological inhibition of CKIδ [22,23] lengthen period and cause arrhythmicity, demonstrating the vital role for DBT/CKI in the clock process. The period-altering mutants of CKI/DBT that do not completely lose kinase activity have more diverse effects on period; DBT^S^, CKIε^tau^ and CKIδ^T44A^ shorten period (only the *dbt^S^* mutation found in Drosophila) whereas DBT^L^, DBT^AR^, DBT^G^ and Dbt^H^ lengthen period (all of these identified in Drosophila). Although these mutations have opposite effects on the clock, they all possess lowered general kinase activity in vitro [7,19,20,24,25].

However, phosphorylation of clock proteins like PER is likely to be more complex in vivo; the period shortening of the CKIδ/ε mutants has been suggested to be due to their ability to phosphorylate PER at some sites more rapidly than wild type CKIδ/ε [26,27], or alternatively from loss of PER phosphorylation at different sites in the period-lengthening and shortening versions of CKI, with some sites destabilizing PER and others stabilizing PER [28]. The different period-altering effects of these mutants might also be due to altered interaction between DBT and other components of the pathway. The amino acid affected by the *tau* mutation has been proposed to contribute to a triad of amino acids that is involved in recognition of previously phosphorylated residues in PER and thereby directs phosphorylation by CKI/DBT to other sites in PER downstream of these phosphorylated residues, or alternatively to affect a nuclear localization signal [19]. We carried out mutations in and around these proposed mediators of phosphate recognition or nuclear localization and expressed these proteins in flies, to see if they altered period similar to the *tau* mutation in vivo. Mutations affecting lysines or arginines in either the phosphate recognition triad, the NLS or a lysine outside either the phosphate recognition triad or the NLS all shortened circadian period, while mutations of internally located arginines lengthened circadian period. These results suggest that a surface domain including the NLS and phosphate recognition motif can be mutated to shorten circadian period and raise the possibility that disruption of a DBT protein-protein interaction produces the effect. However, only some of the mutations disrupted the previously demonstrated DBT/BDBT interaction [29] while the tau mutation did not, so the domain is predicted to disrupt interactions with another protein to produce the short-period rhythms.

## 2. Results

### 2.1. Mutations of Lysines that Are Part of or Close to the Proposed Phosphate-Binding Triad and DBT NLS Cause Short Periods When Expressed with the timGAL4 Driver

**Scheme 1:**
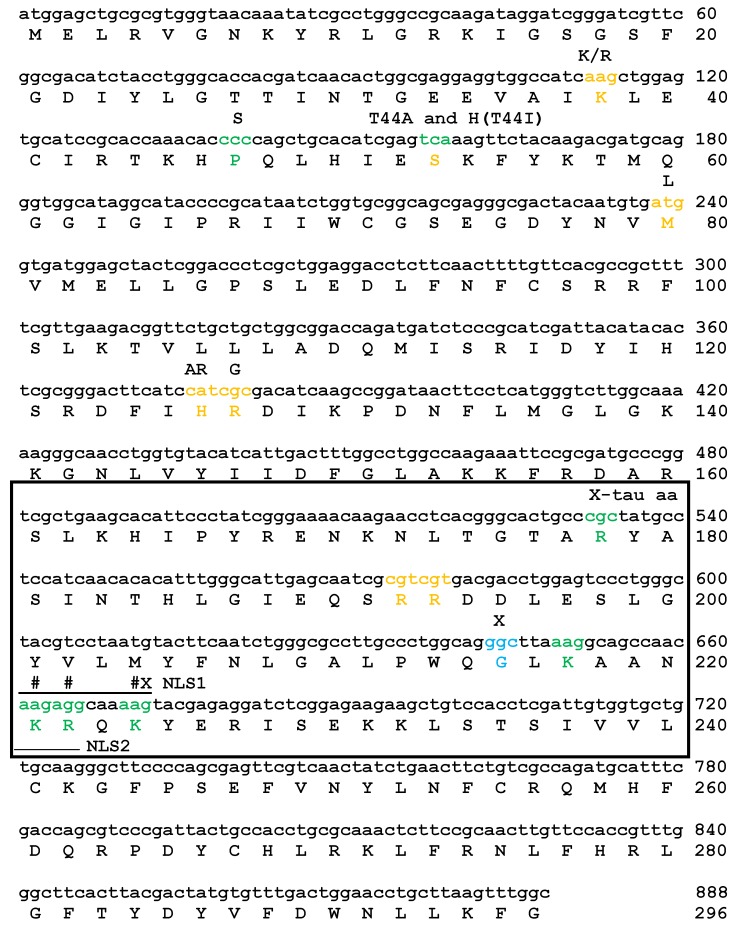
dbt mutations discussed in the text. The nucleotide sequence and corresponding primary amino acid sequence are shown, with numbers of the terminal residue indicated on the right. Amino acid changes that produced period lengthening are colored orange while those that produced period shortening are colored green (note that T44 can be mutated to cause short periods in humans and long periods in flies). The region mutated in this manuscript is surrounded by a box. Lysines or arginines were changed to asparagine, and the glycine was changed to glutamate. The lysines and arginines in the nuclear localization were changed in groups of three or two as indicated (NLS1, NLS2). Amino acids that are part of the proposed phosphate-binding motif are indicated with an X, while those that are part of the NLS are indicated with a #. The one amino acid that had no effect on period when mutated is indicated with light blue. The amino acid changed in the original dbt^tau^ mutation is indicated (“tau aa”).

These mutations were designed to affect either the phosphate-binding triad (R178, G215 or K224) or the DBT NLS (K221, R222, K224; [19] and Scheme 1)). Lysines or arginines were mutated to asparagine to neutralize their positive charges, so that they would be less likely to bind with negatively charged residues in the phosphorylated substrate or nuclear import machinery. To assess the role of the NLS, our previously published NLS1 [29] was employed with mutation of all three K/R’s to N, and in addition we produced an NLS2 construct in which K224 (which is also part of the phosphate-binding motif) was left unmutated, in order to determine if mutation of the NLS would have effects on period in the absence of any changes in the phosphate-binding residues. If the *tau* mutation produces its short period by disrupting interactions that involve either of these regions, then similar short periods could be produced by mutations in at least one of these NLS mutations.

**Figure 1:**
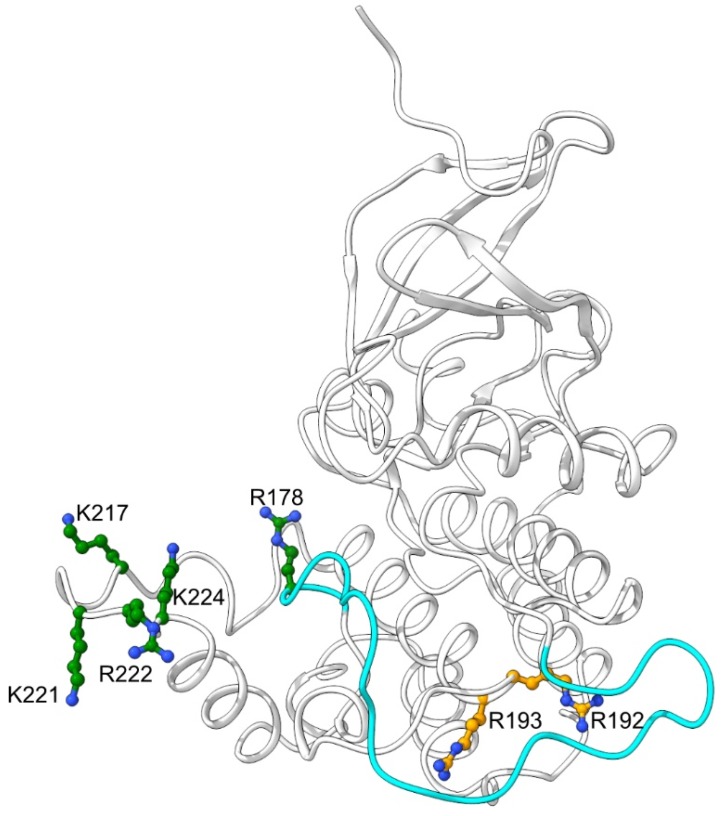
The positions of the amino acids changed in the boxed region of Scheme 1 are indicated in the structure of rat casein kinase Iδ truncated at amino acid 317 (PDB ID 1 CKJ) [30]. Mutations that shortened period are indicated in green, including the tau mutation (R178) and the NLS mutations (K221, R222, and K224). Amino acid changes that lengthened period (R192 and R193) are colored orange. The aliphatic portions of these amino acids are buried and located below the activation loop (colored cyan on the figure). Mutations of these amino acid residues to asparagine presumably alter the position of the activation loop, leading to an increase in period. This figure was prepared using Chimera X [31].

All of these mutated genes were inserted site-specifically at the attP2 locus on chromosome III and were expressed with the timGAL4 driver in circadian cells. Our previous analyses have shown that this overexpression protocol prevents endogenous DBT from interacting significantly with PER and produces the period length of the overexpressed transgene, including a 21.5 h period for flies overexpressing the DBT^Tau^ protein [10,21]. The positions of these amino acid changes in the three-dimensional structure of CKIδ are indicated in Figure 1.

**Figure 2:**
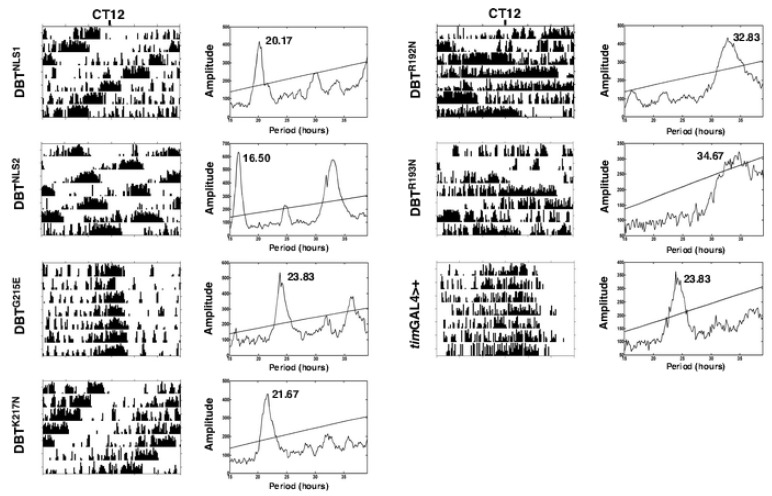
Representative circadian activity records. Males from lines with the indicated DBT transgene were crossed to timGAL4 virgins, and male progeny with the indicated genotypes (timGAL4/+> UAS-DBT/+ or timGAL4/+> +/+) were assayed for locomotor activity in constant darkness. The actograms and periodograms for a representative fly of each genotype are shown. CT12 indicates time of lights off in the previous 12 h:12 h LD cycle.

**Figure 3:**
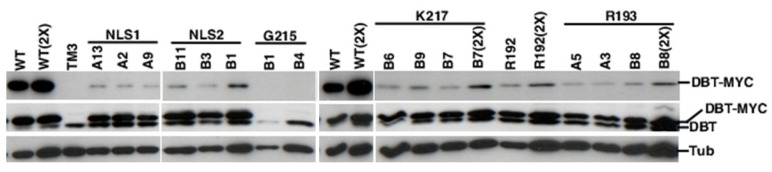
Analysis of transgenic and endogenous DBT levels in the heads of flies collected at ZT7. Extracts from flies expressing the various MYC-tagged DBT proteins with timGAL4 were subjected to SDS-PAGE on 10% gels, blotted to nitrocellulose and probed with anti-MYC antibody (top strip, to detect transgenic DBT), anti-DBT (middle strip, to detect both endogenous and transgenic DBT), and anti-tubulin. The amount of antigen was detected with chemiluminescence. “WT” was wild type DBT expressed from a DBT transgene inserted at another locus (not the attP2 site), and TM3 flies acquired the TM3 balancer rather than the responder transgene. 2× lanes obtained twice the amount of extract as the lane to the left.

Both the NLS1 and NLS2 mutations produced short circadian periods (Table 1 and Figure 2). The NLS2 mutation produced shorter periods (16.5–17 h) than either NLS1 mutation (19.6–22.4 h) or the *dbt^tau^* mutation (21.5 h; see also [21,29]), showing that mutation of the phosphate-binding K224 was not needed for the shortened period (in fact, presence of this mutation lengthened period in the context of the K221N and R222N). As another test of the phosphate-binding triad, another residue that is part of this triad (G215) was mutated to glutamate, with the premise that a negative charge would disrupt binding to any phosphate residues in PER. This mutation did not alter period (Table 1, Figure 2) but the level of expression of this protein was not detectable (Figure 3), suggesting that it affected protein stability and hence caused no change in the period.

Although localization of NLS1 mutant DBT in adult brains was cytoplasmic, applying this mutation to the DBT^K/R^ eliminated its capacity for dominant negative effects on circadian rhythms as well as its interaction with BDBT, and the addition of a strong NLS sequence to DBT^WT^ did not alter period, suggesting that the altered localization of DBT is not the reason that these mutations shorten period [29]. The other lysine between the *tau* amino acid and the NLS was mutated to asparagine, and this K217N mutation also led to a shortening of period similar to that of the *tau* mutation (DBT^tau^—21.5 h; DBT^K217N^—21.6–22 h; Table 1 and Figure 2). Therefore, mutations of lysines outside the NLS also can lead to short periods.

### 2.2. Mutations that Affected Arginines Localized Internally Lengthened Period

There are two other positively charges residues lying between the *tau* amino acid (K178) and the NLS, but these residues (R192 and R193; Scheme 1) are localized internally rather than on the surface (Figure 1) and could potentially affect protein structure and activity. We analyzed two more mutations in these amino acids (R192 or R193). As with the other mutations of lysines or arginines, these were mutated to asparagine. Mutations of both the arginine residues led to a lengthening of period (33.2–34.9 h; Table 1 and Figure 2) rather than a shortening of period. This different effect on period was consistent with our hypothesis that the region around the *tau* mutation on the surface of the protein is probably involved in interactions with other participants of the clock mechanism and hence alters the period in a similar way by shortening it, without altering the overall structure of DBT needed for its catalytic activity.

### 2.3. All of the K/N or R/N Mutant Proteins are Expressed at Comparable Levels, While the G215E Protein is not Detectably Expressed

We analyzed the levels of expression of transgenic and endogenous DBT in these mutants to see whether a change in levels can account for the different periods produced (Figure 3). All but one of the constructs expressed DBT at comparable levels. While detection of the mutant DBTs was less than that of DBT^WT^ with an anti-MYC antibody (top panel), the detection of DBT with an antibody to the C terminus of DBT did not differ between wild type and mutant DBTs (middle panel), so the period-altering mutations in this region are somehow altering the interaction of the MYC or C terminal domain epitope with the antibody. Potentially, the wild type DBT may phosphorylate its C terminal domain more extensively and reduce the capacity of the anti-DBTC antibody to interact with it. In any event, differences in expression cannot explain the differences in periods (ranging from very long to very short). As noted earlier, G215E mutant protein resulted in a loss of DBT detection, suggesting that the stability of this protein was affected, and hence there was no change in period of this mutant because it could not compete with endogenous DBT. The middle panel in Figure 3 was produced with an antibody that detects both endogenous DBT and transgenic DBT-MYC (slower mobility), and in all cases the transgenic protein is expressed at higher levels than the endogenous DBT in the cells where they are co-expressed, particularly since the DBT-MYC is only expressed in the timGAL4 cells, while endogenous DBT is likely to be expressed more broadly and yet has a lower signal.

### 2.4. The DBT NLS and R192N Mutant Proteins are Defective in Interactions with BDBT

**Figure 4:**
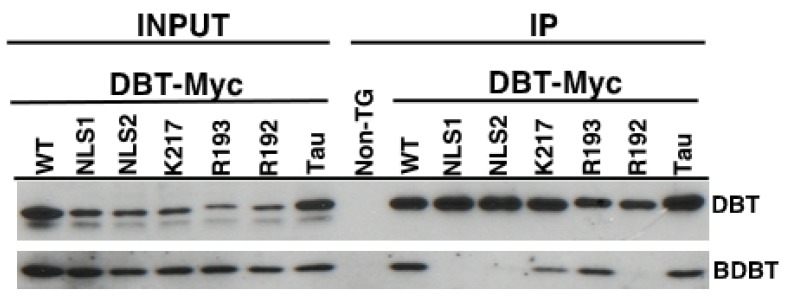
Co-immunoprecipitation analysis of DBT-MYC and BDBT. The indicated transgenic proteins were expressed in flies with the timGAL4 driver, heads were collected at ZT7, extracts prepared, and DBT-MYC was immunoprecipitated with an anti-MYC antibody. In an immunoblot analysis, DBT was detected in the crude homogenates (input) and IPs with an anti-DBTC antibody (top), and BDBT was detected in the same samples with an anti-BDBT antibody (bottom). The DBT^TAU^ line has been previously described [10,21] and shows co-immunoprecipitation of DBT and BDBT along with the K217N and R193N constructs. Non-TG = non-transgenic (no MYC tag on DBT). The result is representative of six replicates with different sample collections.

We previously showed that the DBT NLS1 mutation disrupts the ability of BDBT to interact with DBT [29]. Here, we show that the DBT NLS2 and the R192N mutations also disrupt the interaction of BDBT in co-immunoprecipitations with MYC-tagged DBT from fly heads, but the DBT K217N, DBT R193N and DBT^tau^ protein do not disrupt this interaction (Figure 4). Therefore, the lack of an interaction with BDBT is not necessary to produce a short period. We also assessed pull-downs of PER with DBT-MYC but were not able to detect PER signals, most likely because of a weak PER interaction and degradation during the co-IP (cleaved PER bands were detected in the inputs).

## 3. Discussion

DBT has been shown to play a major role in circadian rhythms by altering the phosphorylation-dependent stability of PER and thus regulating the feedback loop that confers the molecular oscillations in the clock cells. DBT plays a dual role on PER in control of this core mechanism (i) by relieving the effects of PER on the transcriptional repression when it targets nuclear PER for degradation and (ii) by delaying PER’s nuclear localization when targeting its degradation in the cytoplasm. Although the period shortening mutants of DBT (DBT^S^ and DBT^tau^) mutants have been shown to have opposite effects on the period from the period lengthening mutations (DBT^L^, DBT^AR^, DBT^G^ and DBT^H^) [5,7,10,21], they all possess lower kinase activity in vitro [7,19,24,25]. It has been proposed that the effects of these mutations may reduce the intrinsic DBT kinase activity at different PER sites, some of which lengthen and some of which shorten circadian period when phosphorylated [6,28,32]. The tau mutation has also been suggested to be a gain of function mutation that more rapidly phosphorylates certain sites of PER, thus leading to accelerated degradation of the nuclear PER [26,27].

However, DBT protein clearly affects other aspects of clock function besides PER stability. The *dbt^S^* mutation does not affect PER degradation kinetics [33] and has been shown to delay nuclear accumulation of PER protein and affect the feedback regulation of *per* mRNA [8], and it remains uncertain whether DBT kinase activity promotes or antagonizes PER nuclear localization [8,10,34,35]. DBT has also been suggested to be essential for the repressor activity of PER, since the absence of DBT protein leads to decreased repression by PER of its own transcription [35]. Intriguingly however, PER’s repression is present if a catalytically impaired or inactive DBT is present [34,36], suggesting that DBT’s kinase activity does not directly produce repression but may contribute to recruitment of other clock proteins which mediate PER’s repression [36]. DBT also targets CLK [36,37] for phosphorylation, which acts as another control point in the clock mechanism, although CLK is targeted by the catalytically inactive DBT^K/R^, suggesting that DBT targets another kinase to CLK [36]. Because DBT is part of a clock protein complex that contains other proteins in addition to PER, some of these effects may be mediated by DBT’s protein/protein interactions with other clock complex components, rather than directly as a consequence of its kinase activity.

It has been proposed that *tau* amino acid may affect part of the CKI/ε phosphate binding/recognition site that was suggested to preferentially target CKIε to phosphorylate PER downstream from previously phosphorylated sites, or alternatively that it interacts electrostatically with an ion that also affects the NLS of CKIε. One of the amino acids mutated in DBTNLS1 (K224) was indeed a part of the phosphate-binding site, but the DBTNLS2 mutant protein does not include a mutation of this amino acid but does include mutations of other amino acids in the NLS, in order to determine if these mutations produce a short period. In fact, this NLS2 mutation produced a period that was even shorter than mutation of the entire NLS. Alternatively, we also mutated an amino acid that was not part of either the phosphate-binding domain or the NLS to see if short periods might be produced, and the K217N mutation did produce a short period rhythm. Mutations of the R192N or R193N lengthened period, showing that mutations of internally placed amino acids can have an opposite effect on period. In fact, these residues are located below the activation loop, of which the tau residue is also part (Figure 1). Mutations of these amino acids presumably alter the position of the activation loop, leading to an increase in period. The capacity of K217N, K221N and R222N mutations to shorten circadian period means that the effective residues extend outside the residues directly involved in binding of a negative ion in the crystal structure, and in fact only the original *tau* mutation affects the phosphate-binding triad, making disruption of the NLS more supported by our current studies for the period-shortening effects than disruptions of the phosphate-binding triad (Scheme 1). However, our current studies have not directly addressed the role of K224 and G215 in period shortening, so the role of the putative phosphate interaction domain has not been as directly addressed. In addition, the K217N mutation effect extends the domain beyond the NLS. Of course, it is possible that these mutations are close enough to the NLS and the phosphate-binding amino acids to affect phosphate binding or nuclear localization. Moreover, binding of a phosphorylated substrate to the phosphate-binding site may alter the interactions or conformation of the NLS, affecting its capacity to interact with other factors, and likewise binding of the NLS to nuclear import factors may affect the capacity of the enzyme to interact with phosphorylated residues. Therefore, the function of this region of the kinase may allow an integrated response to nuclear localization and catalytic activity. Nevertheless, it is unlikely that the previously observed enhanced cytosolic localization of DBT NLS1 protein [29] can explain the shorter circadian period, as enhanced DBT activity in the cytosol should delay the daily accumulation of PER and slow down the circadian clock rather than expedite it. It is more likely that these period-shortening mutations disrupt interactions of other proteins in addition to nuclear import factors. It is interesting that the NLS and R192N mutations disrupt BDBT binding, while the other short-period mutations in this region do not. As reductions in BDBT lead to longer periods rather than shorter periods [38], it is unlikely that the short periods arise from reductions in BDBT binding. Likewise, reductions in the other DBT interactor we have identified (SPAG) lengthen period [39], again making it unlikely that the short periods arise from reductions in SPAG binding. Potentially the short periods could involve disruptions of binding of one or more factors that remain undiscovered.

The disrupted binding of DBT with its interactor could contribute to altered PER phosphorylation consistent with either of the models so far proposed—reduced phosphorylation at different sites for shortening and lengthening CKI alleles or site-specific gain of function at particular PER sites. In fact, disruptions of binding to PER itself could alter PER phosphorylation in this manner. However, as noted above, there is also evidence that DBT recruits other factors needed for PER and CLK phosphorylation and repression, some of these effects do not require its kinase activity, and the *dbt^S^* mutant does not affect PER degradation to shorten period. Recent evidence has argued that mammalian CKIδ has evolved a different kinase capacity for serine-rich motifs from Drosophila DBT [40], suggesting that the catalysis of Drosophila DBT and mammalian CKIδ may show major differences. And yet, the effect of the *tau* mutation is conserved in mammals and flies in both CKIε and DBT [19,21], consistent with an effect on an evolutionarily conserved aspect of CKI function, perhaps independent of its catalytic activity.

Based on the above evidence, we can conclude that we have uncovered a domain of DBT we have termed “TAU domain”, which shortens period when mutated. The domain includes the NLS but is slightly larger (including K217), extends outward from the rest of the protein (Figure 1, green), and therefore may affect interactions with regulators besides or in addition to substrates and importins. Clearly, BDBT is one interactor that is affected, but the effects on BDBT do not explain the period shortening effects. Since the “TAU domain” mutations that shorten the period are located in close proximity on the surface and the mutations that lengthen period are located inside the protein, it is likely that these period-shortening mutations may alter the interaction of DBT with other factors in addition to BDBT, perhaps all of which interact with this region as part of a multi-protein complex.

## 4. Materials and Methods

### 4.1. Site-Directed Mutagenesis

For mutational analysis of nuclear localization and the phosphate-binding domain region, the pMT-DBT^WT^- MYC plasmid [24] was used to generate different mutants with the QIAGEN QUIKCHANGE (Stratagene CA) using the following primers. The mutations were confirmed by DNA sequencing. The list of mutants and the oligonucleotide primers are as follows (mutated codons underlined):

DBT R192N Forward, 5′-GGGCATTGAGCAATCGAACCGTGACGACCTGGAGTC;
DBT R192N Reverse, 5′-GACTCCAGGTCGTCACGGTTCGATTGCTCAATGCCC;
DBT R193N Forward, 5′-GCATTGAGCAATCGCGTAACGACGACCTGGAGTCCCTG;
DBT R193N Reverse, 5′-CAGGGACTCCAGGTCGTCGTTACGCGATTGCTCAATGC;
DBT G215E Forward, 5′-CGCCTTGCCCTGGCAGGAATTAAAGGCAGCCAACAAG;
DBT G215E Reverse, 5’-CTTGTTGGCTGCCTTTAATTCCTGCCAGGGCAAGGCG;
DBT K217N Forward, 5’-CTGGCAGGGCTTAAACGCAGCCAACAAGAGG;
DBT K217N Reverse, 5’-CCTCTTGTTGGCTGCGTTTAAGCCCTGCCAG;
DBT NLS2 Forward, 5’-GCTTAAAGGCAGCCAACAACAACCAAAAGTACGAGAGGATC;
DBT NLS2 Reverse, 5’-GATCCTCTCGTACTTTTGGTTGTTGTTGGCTGCCTTTAAGC;
DBT NLS1 Forward, 5’-GCTTAAAGGCAGCCAACAACAATCAAAACTACGAGAGGATCTCG;
DBT NLS1 Reverse, 5’-CCGAGATCCTCTCGTAGTTTTGATTGTTGTTGGCTGCCTTTAAGC.

### 4.2. Generation of Transgenic Flies with Mutant UAS-DBT Proteins Expressed in Circadian Cells

Transgenic constructs were made by using the Φc31 integrase system [41]. The pUAST-attB polylinker was first modified by inserting a PmeI site. This site was introduced by digesting the plasmid with NotI and XhoI, followed by ligation of a double-stranded oligonucleotide produced by annealing two single-stranded oligonucleotides, 5′-GGCCGCCCAAGGTTGTTTAAACC-3′ and 5′-TCGAGGTTTAAACAACCTTGGGC-3′. Successful insertion of the oligonucleotide created a PmeI site in the pUAST-attB polylinker, which was consecutively digested with PmeI and EcoRI to allow oriented insertion of PmeI-EcoRI-digested fragments obtained from our various pMT-DBT-MYC constructs. This placed the DBT gene under control of the upstream activation sequence (UAS) promoter. Fly transformants were produced by model system genomics of Duke University (Durham, NC, USA) at the attP2 locus on chromosome III [41]. The lines were generated and were all mapped by standard procedure to the third chromosome. The lines were balanced with TM3SbSer, a third chromosome balancer.

Males from one or more independently derived lines were crossed to timGAL4 virgins (line 7126 from the Bloomington stock center, University of Indiana, Bloomington, IN, USA), and male progeny were collected for analysis of circadian rhythms.

### 4.3. Analysis of Circadian Locomotor Activity Rhythms

Male flies placed into glass cuvettes were assayed in behavioral monitors from Trikinetics (Waltham, MA, USA), with 10 min collection bins. The flies were entrained for at least three days to LD 12 h:12 h and then monitored in constant darkness for 7 or more days. Individual actograms were obtained for all flies, and ClockLab was used to perform chi-square periodogram analysis to determine rhythmicity and period length. Rhythmic flies produced single statistically significant peaks, and the period length of the peak was taken as the period for each fly.

### 4.4. Analysis of Transgenic and Endogeonous DBT Levels

Fly heads were cut off by razor blade from 5 flies from each genotype at ZT 7, homogenized in 1.1× Laemmli SDS buffer, heated to 100 °C for 5 min, and stored at −80 °C until analysis. The extracts were subjected to SDS-PAGE on 10% polyacrylamide gels, transferred to nitrocellulose, and probed with anti-DBTC [10], anti-MYC (ThermoFisher catalog# 13-2500, Waltham, MA, USA), anti-BDBT [38], or anti-tubulin (Biolegend catalog # 903401, San Diego, CA, USA). Signals were obtained in response to the appropriate HRP-coupled secondary antibody (American Qualex, San Clemente, CA, USA) and ECL-Plus detection methods. Images were obtained on X-ray film.

### 4.5. Immunoprecipitations to Measure DBT/BDBT Interactions

Fly heads were cut off by razor blade from 100 flies from each genotype collected at ZT7, homogenized, treated with mouse anti-MYC IgG and gamma-bind beads, centrifuged and washed as previously described [38]. The crude extracts and immunoprecipitates were then subjected to immunoblot analysis with rabbit anti-DBTC and guinea pig anti-BDBT [38].

## Figures and Tables

**Table 1:** Effects of Mutations Around the DBT NLS and Phosphate-Binding Domain.

DBT	Line	Genotype	Avg. Period (h)+SEM	%Rhythmicity (N)
DBT^NLS1^	A2	timGAL4>UAS-*dbt*	20.3 ± 0.1 ^a^	93 (14)
	A9	timGAL4>UAS-*dbt*	22.4 ± 0.2 ^b^	93 (14)
	A13	timGAL4>UAS-*dbt*	19.6 ± 0.2 ^a^	83 (12)
DBT^NLS2^	B1	timGAL4>UAS-*dbt*	16.6 ± 0.05 ^c^	100 (15)
	B3	timGAL4>UAS-*dbt*	16.5 ± 0.08 ^c^	100 (16)
	B11	timGAL4>UAS-*dbt*	17.0 ± 0.07 ^c^	87.5 (16)
	B13	timGAL4>UAS-*dbt*	16.6 ± 0.03 ^c^	94 (16)
DBT^G215E^	B1	timGAL4>UAS-*dbt*	23.9 ± 0.1^d^	100 (25)
	B4	timGAL4>UAS-*dbt*	24.0 ± 0.2^d^	100 (12)
DBT^K217N^	B6	timGAL4>UAS-*dbt*	21.6 ± 0.1 ^b^	100 (15)
	B7	timGAL4>UAS-*dbt*	22.0 ± 0.2 ^b^	93 (15)
	B9	timGAL4>UAS-*dbt*	21.9 ± 0.08 ^b^	100 (15)
DBT^R192N^		timGAL4>UAS-*dbt*	33.2 ± 0.02	80 (15)
DBT^R193N^	A3	timGAL4>UAS-*dbt*	34.0 ± 0.4 ^e^	87.5 (16)
	A5	timGAL4>UAS-*dbt*	34.9 ± 0.5 ^e^	100 (7)
	B8	timGAL4>UAS-*dbt*	34.1 ± 0.3 ^e^	94 (16)
DBT^+^		timGAL4>+	24.3 ± 0.2 ^d^	93 (14)

Residues in DBT were mutated via site-specific mutagenesis as shown in Scheme 1. The mutated responder transgene linked to a GAL4-responsive UAS sequence was integrated site-specifically into the Drosophila genome via phiC31-mediated integration, with multiple lines for each construct produced by separate integrations. LD 12 h:12 h-entrained male progeny hemizygous for both the timGAL4 driver and responder were placed in locomotor assays in constant darkness (DD). Rhythmic flies produced single strong peaks in the periodogram analysis. The mean period ± standard error of the mean (SEM) and the percentage of rhythmicity (N, number of flies for line averages) are indicated for each line. A one-way ANOVA of individual fly periods showed a statistically significant effect of genotype on periods (F(16, 221) = 1274.1, *p* < 0.0001). Genotypes of flies whose average periods did not differ significantly by a Tukey HSD test (*p* > 0.03) are identified with the same letter. A Kruskal-Wallis ANOVA with multiple comparisons of mean ranks for all genotypes did not identify any statistically significant differences in the % rhythmicity (H(16, N = 253) = 17.0, *p* = 0.39).
